# Karyomaps of cultured and cryobanked *Litoria infrafrenata* frog and tadpole cells

**DOI:** 10.1016/j.dib.2018.04.025

**Published:** 2018-04-13

**Authors:** Richard Mollard

**Affiliations:** Faculty of Veterinary and Agricultural Science, University of Melbourne, Parkville 3052, Australia

## Abstract

These data and analyses support the research article “Culture, cryobanking and passaging of karyotypically validated native Australian amphibian cells” Mollard (2018) [1]. The data and analyses presented here include: (1) three additional karyomaps of cells from the cryobanked and passaged frog and tadpole species *Litoria infrafrenata*; and (2) combined short-to-long arm ratios of the four karyomaps measured from each respective animal here and in Ref [1].

**Specifications table**

**Table:** 

Subject area	*Biology*
More specific subject area	*Cryobiology and conservation*
Type of data	*Figures and table*
How data was acquired	*Microscope: Olympus BX60 microscope, colour CCD Leica DFC425C camera, and EL-6000 Leica light source*
Data format	*Analysed*
Experimental factors	*Cell cultures were treated with colcemid, stained with DAPI and coverslipped in Gelvatol mounting medium*
Experimental features	*Karyotypes of Litoria infrafrenata frog and tadpole cells were determined after culture, freeze-thawing and passaging for expansion. Chromosomes were paired and ordered according to length. Long-to-short arm ratio measurements of each karyotype were compiled to give average measurements for chromosome type designation (e.g. metacentric type).*
Data source location	*Not applicable*
Data accessibility	*Data is with this article*

**Value of the data**

These data are of value to the scientific community for the following reasons:

● These data demonstrate reproducibility of karyotypes determined following culture and cryostorage of *Litoria infranfrenata* tadpole and frog cells, and
● These data demonstrate the measured short-to-long arm ratios of each chromosome and provide designation of metacentric, submetacentric and subtelocentric to each chromosome, thus permitting direct comparisons to chromosomal karyomaps from living animals of the same species [1], [2], [3].

## 1 Data

**Fig. 1:**
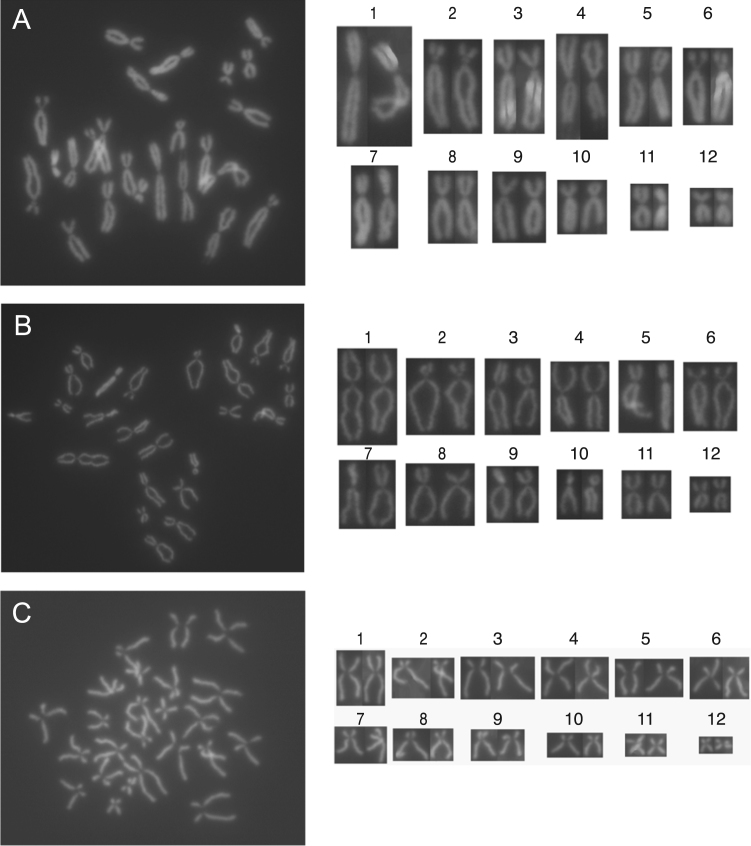
*Litoria infrarenata* frog karyomaps. (A, B and C) Following culture, freezing and thawing, three further examples of the passage *2 Litoria infrafrenata* karyotype confirmed the 2 N = 24 diploid chromosome. Chromosome 1 is larger, chromosomes 2 to 9 are larger to medium, and chromosomes 10 to 12 are smaller in size.

**Fig. 2:**
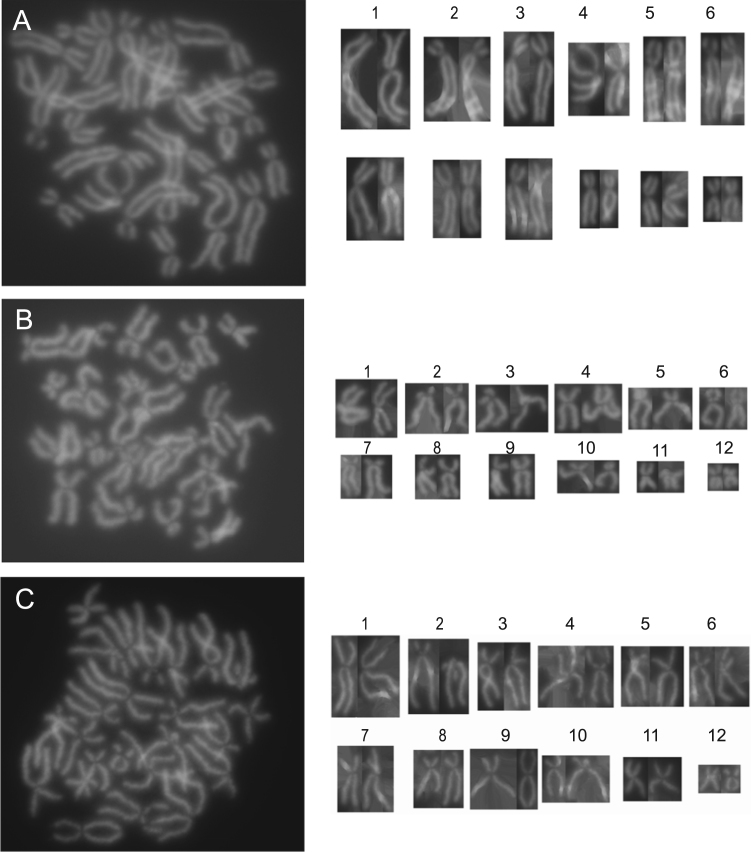
*Litoria infrarenata* tadpole karyomaps. (A, B and C) Following culture, freezing and thawing, three further examples of the passage *3 Litoria infrafrenata* tadpole karyotype confirmed the 2 N = 24 diploid chromosome number. Chromosome 1 is larger, chromosomes 2 to 9 are larger to medium, and chromosomes 10 to 12 are smaller in size.

The presented data were obtained following DAPI staining of metaphase spreads of cultured, cryobanked and recultured *Litoria infrafrenata* frog and tadpole cells. *Litoria infrafrenata* frog cell chromosomes 1, 3, 5, 6, 7, 8, 9 and 10 are designated as submetacentric, while chromosome 2 is designated subtelocentric and chromosomes 4, 11 and 12 are designated metacentric (Fig. 1 and Table 1). *Litoria infrafrenata* tadpole cell chromosomes 1, 4, 11 and 12 are designated metacentric, chromosome 2 is designated subtelocentric, and 3, 5, 6, 7, 8, 9 and 10 are designated submetacentric (Fig. 2 and Table 1). A two tailed, type 2, Student's t-test demonstrates no significant difference between the short-to-long arm ratios of the frog and tadpole chromosomes 1 (*p* = 0.30). The frog chromosome 1 is borderline metacentric/submetacentric while the tadpole chromosome 1 is metacentric.

**Table 1:** Chromosome short arm to long arm ratios. Values of short arm to long arm ratios are given as the average of four prepared and measured karyomaps ± standard deviation. *Litoria infrarenata* frog and tadpole chromosome 1 is borderline metacentric/submetocentric for the frog assayed and metacentric for the tadpole assayed, chromosomes 3, 5, 6, 7, 8, 9 and 10 are submetacentric, chromosome 2 is subtelocentric and chromosomes 4, 11 and 12 are metacentric.

	**Chromosome number**			
	1	2	3	4
*L. infrafrenata* (frog)	1.7 ± 0.3	3.8 ± 0.5	2.2 ± 0.5	1.2 ± 0.1
	Submetacentric	Subtelocentric	Submetacentric	Metacentric
				
*L. infrafrenata* (tadpole)	1.6 ± 0.4	3.8 ± 0.9	2.8 ± 0.4	1.6 ± 0.4
	Submetacentric	Subtelocentric	Submetacentric	Metacentric
	
	**Chromosome number**			
	5	6	7	8
				
*L. infrafrenata* (frog)	2. 3 ± 0.8	2.5 ± 0.4	2.1 ± 0.4	2.5 ± 0.6
	Submetacentric	Subtelocentric	Submetacentric	Metacentric
				
*L. infrafrenata* (tadpole)	2.3 ± 0.7	2.6 ± 0.1	2.6 ± 0.9	2.3 ± 0.7
	Submetacentric	Subtelocentric	Submetacentric	Metacentric
	
	**Chromosome number**			
	9	10	11	12
				
*L. infrafrenata* (frog)	2.2 ± 0.3	2.6 ± 0.6	1.5 ± 0.3	1.5 ± 0.3
	Submetacentric	subtelocentric	Submetacentric	Metacentric
				
*L. infrafrenata* (tadpole)	2.0 ± 0.4	2.4 ± 0.5	1.4 ± 0.2	1.6 ± 0.4
	Submetacentric	subtelocentric	Submetacentric	Metacentric

## 2 Experimental design, materials and methods

For karyotyping cells were treated for six to eight hours with 0.1 μg/ml KaryoMAX® colcemid (GIBCO) and then stained with 40,60-diamino-2-phenylindole (DAPI; 500 ng/ml; Sigma) according to manufacturer's instructions and as previously described [4]. Slides were prepared by conventional drop-splash technique and coverslipped with DAPI in Gelvatol mounting medium [5]. The largest chromosome was designated chromosome 1, and the remaining were designated following descending chromosomal length [2], [3], [6]. Chromosome arms were measured using the Levan plugin on Image J software [7]. Chromosomal designation as metacentric, submetacentric or subtelocentric, respectively, were defined as: 1 – 1.69, 1.7 – 2.99 and 3 – 6.99, long arm to short arm ratios, respectively [6]. Imaging was performed under oil immersion at 1000 × using an Olympus BX60 microscope, colour CCD Leica DFC425C camera, and an EL-6000 Leica light source. Photographs of DAPI stained karyotypes were captured using Leica LAS-AF and Q-Capture Pro7 Version 7.0.5 Build 4325 software (QImaging Inc, USA).
