# Supplementary material for: Karyomaps of cultured and cryobanked Litoria infrafrenata frog and tadpole cells
Source: Data Brief. 2018 Apr 13;18:1372–7. doi: 10.1016/j.dib.2018.04.025 (PMC5997010; doi:10.1016/j.dib.2018.04.025)
Supplement: Supplementary file 1 — Supplementary material [file mmc1.docx]

**CONFLICT OF INTEREST**

The author is the owner of Amphicell Pty Ltd, an Australian native frog conservation advocacy aiming to safeguard the future of amphibian biodiversity within Australia.
